# “Do It by Myself” or Autonomy, Participation, and Assistive Devices and Technology Needs of Children and Youth With Disabilities: Text Mining Analysis of a National Survey in France

**DOI:** 10.2196/90708

**Published:** 2026-07-08

**Authors:** Johanne Mensah Gourmel, Maxime Bourgain, Maxime Galloy, Mario Veruete, Gwenaël Cornec, Sylvain Brochard, Christelle Pons, Arriel Benis

**Affiliations:** 1 Physical Medicine and Rehabilitation Department, Centre Hospitalier Régional Universitaire de Brest, 2 Avenue Maréchal Foch, Brest, 29200, France, 33 298223152; 2 INSERM UMR 1101, Université Brest, Brest, France; 3 Pediatric rehabilitation department, Fondation Ildys, Brest, France; 4 Arts et métiers Institute of Technology, EPF Engineering School, institut de biomécanique humaine Georges-Charpak (IBHGC), université Sorbonne Paris Nord, Paris, France; 5 EPF - École d'ingénieurs, Cachan, France; 6 Coruscant, Applied Artificial Intelligence Division, Willing Technologies, , Toulouse, France; 7 Department of Biomedical Engineering, Duke University, Durham, NC, United States; 8 Department of Biomedical Informatics, Jacobs School of Medicine and Biomedical Sciences, University at Buffalo, Buffalo, NY, United States

**Keywords:** text mining, surveys and questionnaires, children and youth with disabilities, individuals with disabilities, community participation, activities and participation, assistive technology, France

## Abstract

**Background:**

A 2019 French national online survey explored activity limitations and participation restrictions among children and youth with disabilities for which innovative solutions could help.

**Objective:**

The primary aim of this study was to use text mining analysis to complement previous human-led qualitative research and to uncover actionable additional knowledge related to difficult life situations that innovative solutions could help address and to compare the perspectives of children and youth with disabilities, relatives, and professionals.

**Methods:**

This study was part of *Innovation for Participation*, a cross-sectional online survey, including (1) closed questions about sociodemographic characteristics, (2) closed questions about life situations that induced difficulties, and (3) open questions to explore these situations in greater depth. This study used a text mining approach designed for French data to analyze answers to open-ended questions about difficulties and desired innovative solutions. The analysis process included (1) data cleansing; (2) computing and analyzing the word frequency in the answers and their cooccurrence; (3) clustering answers using K-means algorithms; (4) analyzing the cluster words to define a title for each one and identify answers that are consistent with cluster titles, thereafter defined as well-classified; and (5) evaluating internal consistency defined as the ratio of well-classified answers to the total number of answers.

**Results:**

In total, 1055 responses were received, and 962 (91.2%) were included, representing 2018 open-ended answers to questions about difficult life situations for which innovative solutions could be useful. Frequency analysis highlighted the desire for action and autonomy by children and youth with disabilities and their relatives (“do” in first position, closely followed by “by themselves” in third position). “Disability” was frequently used by professionals (third position) but did not figure among the 20 most frequently used terms by other respondents. Cooccurrences with “do” revealed a deep interest in “doing sports.” Answers were gathered into 14 clusters, ranging in size from 9 to 681 answers; 5 underwent a second clustering step. Internal consistency ranged from 42% to 100%. Finally, 8 clusters were related to the difficulties linked to a desire for autonomy, and 4 highlighted difficulties related to actions within a group or community. Some clusters reinforced domains that emerged in the human-led analysis.

**Conclusions:**

Analysis of 2018 open-ended answers using a text mining approach designed for French data revealed that solutions are required to facilitate autonomy and the ability to “do it by myself (or themselves),” as well as integration into group and community life. Complementary to previous human-led analysis, the results reinforced the need for solutions to facilitate mobility, transport, and sports. They additionally highlighted differences in vocabulary used between the 3 respondent groups, suggesting that professionals may need to consider the language they use when communicating with individuals and relatives.

## Introduction

Children and youth with disabilities may encounter activity limitations and participation restrictions [1-3]. These limitations affect children and youth across a broad spectrum of conditions, including physical and motor disabilities, neurodevelopmental conditions (eg, autism spectrum disorder, attention deficit hyperactivity disorder), mental and cognitive disabilities, and sensory impairments. Some difficulties may be overcome using assistive devices and technology [2,4], as defined by the International Classification of Functioning, Disability and Health [5] as any product, instrument, equipment, or technology adapted or specially designed for improving the functioning of a person with a disability. Technological advances have created new opportunities for children and youth with disabilities. Recent literature evidence confirms the potential for assistive technologies whatever the participation domain (eg, school [6,7], leisure, and play [8]) and the origin or type of disabilities (mental or cognitive [9], sensory [6], physical and motor [10], and multiple disabilities [11]) and, more generally, the need for further research and development in these domains. Major challenges remain in the use of technology, including improving access [2,12] and improving design to facilitate adoption [13]. New innovative solutions are still needed when there is no existing satisfying solution [13,14]. As challenges vary considerably in nature and severity across the disability spectrum and therefore developing inclusive technologies that meet the needs of as many varied conditions as possible, a comprehensive understanding of technology needs for new solutions requires engaging all disability types rather than focusing on a single condition.

The user’s opinion is essential in the development of assistive technologies to ensure their relevance [15]. Engaging users (ie, children and youth with disabilities, relatives, and all the professionals involved with children and youth with disabilities in the health care system, at school, and in free-time activities) helps to build a comprehensive picture of their needs [16,17]. Therefore, we developed the “Innovation for Participation” survey to identify activity limitations and participation restrictions for which assistive products and technologies may be useful [18]. The survey was addressed to children and youth with disabilities, relatives, and professionals involved with children and youth with disabilities. Collecting open-ended (and unstructured) opinions may enrich closed-ended answers to questionnaires by allowing participants to freely share their vision; this approach can enable a better and deeper understanding of individuals’ needs [19,20]. Therefore, open-ended questions asked survey participants to describe the difficult life situations mentioned in the closed-ended questions and the desired solutions. In a prior study [18], we analyzed the answers using adapted human-led qualitative methods [21,22]. The results showed that difficulties were frequently highlighted in the closed questions and described in detail in the open questions related to participation in recreational activities, leaving the house and using transport, participating in a group, and getting ready for the day. Transversal explanations for difficulties were provided spontaneously (eg, lack of accessibility and mobility). Solutions proposed included personal assistive devices to facilitate home life, high-tech devices, devices to compensate for impaired body functions, and adapting the familiar environment and daily activities. Few public solutions were proposed. The necessity of human assistance in daily life was emphasized [18].

A recent systematic review showed that methods for analyzing large qualitative datasets still rely on traditional qualitative analyses, such as thematic and content analysis [23]. However, pioneering studies have shown that combining these analyses with text mining can enrich qualitative text analyses [24]. A combined approach may reveal findings not identified by qualitative or text mining analysis alone, thus providing multiple lenses to deepen understanding of text data [25-31].

Techniques such as word frequency analysis can identify the priorities of different groups [32-35] and help to make their voices heard [36]. Classification sorts text data into predefined categories, whereas clustering may facilitate concept emergence by grouping similar answers rather than isolated terms [37,38]. Thus, actionable knowledge may be uncovered without a priori assumptions. For example, a clustering analysis of tweets expressing opinions on vaccination was used to draft a proposal for health policy improvements to enhance vaccination promotion [25]. Similar analyses were applied to prioritize and adapt rare disease associations’ priorities based on user feedback by comparing the relative frequency of words in associations’ priority reference documents with posts on patient Facebook groups [32]. Cooccurrence analysis was used to facilitate communication between patients and health professionals about musculoskeletal disorders, highlighting differences in the concepts used by these groups [36]. Topic modeling of YouTube video posts provided insights into the use of virtual reality tools to raise awareness of Alzheimer disease and related dementia in the general population [38].

Our overall aim was to highlight the insights and experiences of children and youth with disabilities, relatives, and professionals involved with children and youth with disabilities, regarding the real-world challenges they encounter for which a technical solution could be useful. Our objective was to complement a previous human-led qualitative research and to uncover actionable additional knowledge related to difficult life situations for which innovative solutions could be useful [18,25]. Our goals were as follows:

1. To discover actionable knowledge related to difficult life situations for which innovative solutions could be useful, as a complement to findings from the human-led qualitative analysis conducted on the data from this survey
2. To compare the perspectives of children and youth with disabilities, relatives, and professionals involved with children and youth with disabilities. To this end, we analyzed the text data from the “Innovation for Participation” survey [18] using French-language text mining techniques.

Our work was driven by 2 leading hypotheses. The first was that user needs exploration using text mining would enrich our understanding of survey answers by uncovering actionable knowledge, complementing human-led qualitative analysis [18]. The second posited that text mining would effectively reveal user needs while considering the diverse perspectives of people with disabilities, relatives, and professionals. The main challenge of this work was that it was based on a French-language survey for which no language-adapted analytical framework was available.

## Methods

### Ethical Considerations

The study was conducted in compliance with current French regulation (Loi Jardé 2012‐300) [39-41]. It was not within the scope of research involving human subjects; therefore, French law does not require ethical approval for such research protocols [42,43].

### Innovation for Participation Survey

This cross-sectional study was part of “Innovation for Participation,” a national survey aimed at identifying the most frequent activity limitations and participation restrictions for which assistive products and technologies may be useful. It was promoted through the European Academy of Childhood Disability, especially during the 2019 conference, whose leading theme was “Innovation for Participation,” and through the French-Speaking Society for Studies and Research on Disabilities (in French: Société Francophone d’Etudes et de Recherche sur les Handicaps [SFERHE]). It adopted a convergent parallel mixed methods design. An online open survey was developed following the CHERRIES (Checklist for Reporting Results of Internet E-Surveys) [44] guidelines and disseminated to associations, personal, and professional networks in France between March and December 2019 using convenience and snowball sampling methods [18]. The study is reported according to the STROBE (Strengthening the Reporting of Observational studies in Epidemiology) guidelines [45].

### Study Population and Selection Criteria

#### Overview

Three versions were developed to gather the opinions of the included populations, with the aim of providing the broadest possible picture of the lives of children and youth with disabilities:

- Children and youth with disabilities: young individuals with childhood-onset disabilities capable of self-reporting, spanning ages up to 30 years, regardless of the underlying etiology or severity of their impairment (including cognitive, mental, physical, sensory, or multiple disabilities)
- Relatives: family members, legal guardians, or close friends of a child or youth with disabilities
- Professionals: health care, educational, rehabilitation, and leisure or sports professionals actively involved in the care, education, or support of this population.

#### Inclusion Criteria

To be included in the text mining analysis, respondents were required to (1) reside in France at the time of data collection, (2) provide a nonvoid response, and (3) be 30 years of age or younger for the child or youth with disabilities cohort to minimize retrospective memory bias.

#### Exclusion Criteria

Responses were systematically excluded if they were geographical outliers living outside of France, exact technical duplicate entries, completely void responses, or adults with childhood-onset disabilities who were older than 30 years.

These 3 versions can be found online [46-48].

### Survey Description

**Figure 1.**
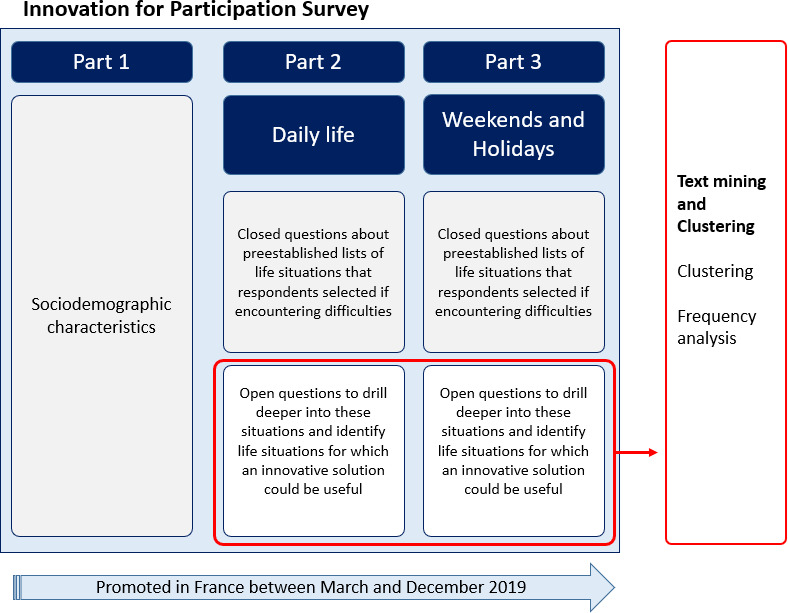
“Innovation for Participation” survey.

The survey included closed and open questions to obtain complementary data on the topic. The closed questions aimed to identify the most frequent life situations for children and youth with disabilities for which a product or technology could be useful for daily life, weekends, or holidays, and the open questions explored these situations in greater depth by asking respondents to describe their personal experiences, to characterize those situations, and to identify desired solutions (Figure 1). The answers to the 4 open-ended questions were analyzed in the study presented here.

The study design, including complementary details about materials and methods, has been described and published elsewhere [18].

### Text Mining Process

To identify priorities for innovation needs and to compare the perspectives of children and youth with disabilities, relatives, and professionals, we carried out a frequency analysis of the data collected. We then conducted a clustering analysis to uncover actionable knowledge from the unstructured data without relying on predefined categories. The study was conducted in 3 stages.

#### Stage 1: Preprocessing and Dataset Cleansing

The answers to the open questions were stored in Microsoft Excel files. As the open questions were not mandatory, void answers were removed.

To ensure accurate text mining analysis, the data underwent initial cleansing. This process involved several key steps: first, all uppercase and accented characters were replaced with lowercase and unaccented characters because omitting accents is a common error among French speakers in writing. Next, numbers and punctuation marks were eliminated. Sentences were tokenized into individual words, then lemmatized to group different inflections under a single root form, allowing for words such as “adapt” and “adapting” to be recognized as the same. More accurately, lemmatization, rather than stemming, was applied using morphological analysis to reduce each word to its dictionary base form rather than simply truncating endings; this approach better preserves semantic accuracy in French, a morphologically rich language. Finally, common stop words (ie, words that occur so frequently that they offer little value for indexing or research purposes) were removed. Although there is no universal list of stop words, existing French-language lists were used and refined with guidance from domain experts.

#### Stage 2: Clustering

Clustering aims at grouping similar answers into a predetermined number of clusters. As the aim was to discover actionable knowledge, clustering was performed on the entire set of answers, including the 3 respondent groups. First, embedding was performed using the numeric TF-IDF (Term Frequency–Inverse Document Frequency) vectorizer. TF-IDF is a statistical measure that weights each word (a.k.a. unigram) in proportion to how often it appears in a given response relative to how common it is across all responses, thereby highlighting words that are distinctive and informative for that response rather than merely frequent everywhere. To quantify this relationship, the TF-IDF weight of a term *t* within a specific document *d* is calculated using the following formula:


$$W_{t,d}={tf}_{t,d}\times log\frac{N}{{df}_{t}}$$

where $W_{t,d}$ is the total weight assigned to the word; ${tf}_{t,d}$ represents the term frequency (TF) or how many times the word appears in this specific document; *N* is the total number of documents in the entire collection analyzed (in this research, the answers to the survey); ${df}_{t}$ being the document frequency or how many different documents contain the specific word *W*; $log(N/{df}_{t})$ represents the inverse document frequency (IDF) and is the “scaling factor” that shrinks the value of common words.

TF-IDF is an embedder that converts each sentence into a vector of numbers, accounting for the occurrence of each term in each answer. As the TF-IDF representation operates at the level of individual words, it captures word-level patterns but does not directly model multiword expressions or sentence-level syntax; this is a known limitation of the approach, discussed further in the *Limitations* section. Semantic algorithms perform relatively well in English, but few are usable in French. Therefore, a numeric embedder was preferred to ensure robustness. Moreover, numeric algorithms group answers by similarities in sentence structures and/or frequent words and word associations, rather than primarily by meaning [37], as is done in qualitative analysis. Therefore, they may help to uncover actionable knowledge. The k-means algorithm was then used to group sentences into k clusters by minimizing intracluster Euclidean distance while maximizing intercluster separation, thereby enabling each word to be interpreted in the context it shares with other words [25,49]. The number of clusters (N) was determined using the Ray-Turi method and index [50]. This method identifies the optimal number of clusters k by minimizing within-cluster compactness while maximizing intercluster separation. When the first minimization was reached, the output consisted of N clusters of vectors and the representative words for each cluster. At this stage, k-means was run with k varying from 2 to 30. For clusters with more than 100 terms, a second clustering step was performed, forming cluster subgroups.

A group of 3 experts (a physician, a health data scientist or medical informaticist, and a biomedical engineer) independently analyzed representative terms and defined a title for each cluster, then met to reach consensus. The obtained clustering was then independently analyzed by 2 researchers (JMG and MB), who annotated each answer for each cluster according to its correspondence (1) or not (0) to the cluster title. When a discrepancy arose between the first 2 researchers, a third researcher (AB) provided his annotation. The internal consistency, defined as the ratio of well-classified answers to the total number of answers, was then calculated. When a second clustering step was performed, the internal consistency was calculated on the subgroups. To verify interoperator consistency, Cohen κ was calculated between the 2 researchers’ annotations. For each cluster, if κ was lower than 0.80, the following process was conducted:

- Knowing that divergences in the calculated internal consistency may be partly due to difficulties in labeling, when the percentage of differently classified answers was above 30%, a new title was proposed, and each researcher performed a new independent classification. Internal consistency was recalculated, and κ was checked again to determine whether the process could proceed to the next step.
- If the title was consensual, discussions were engaged between the 2 researchers on the divergent annotations to reach a consensus (defined by κ values above 0.8) for each item.

#### Stage 3: Frequency Analysis

The obtained text data were 3 corpora of answers. Therefore, frequency analysis was conducted by respondent category using a TF-IDF function to avoid overemphasizing specific terms, as this function weights the frequency of each word based on its occurrence in a single answer relative to its occurrence in the corpus of answers.

An additional function was developed to study word cooccurrence by identifying words frequently associated with a given word in the corpus of answers.

## Results

### Survey Population Characteristics

We received 1005 answers. We excluded answers from respondents who did not live in France (n=32, 3.2%), duplicates (n=4, 0.4%), void answers (n=14, 1.4%), and those from adults with childhood-onset disabilities over 30 years of age (n=53, 5.3%; to avoid memory bias).

Of the 962 included answers, 92 (9.6%) were from children and youth with disabilities (mean age 17.1, SD 5.7 y), 493 (51.2%) were from relatives (mean age 46.6, SD 11.5 y; 378/493, 77.5% were the mother), and 377 (39.2%) were from professionals (mean age 41.3, SD 11.1 y). In total, 764 (79.4%) respondents were women, and 13 (92.9%) of 14 French regions were represented. Regarding the children and youth with disabilities, 81.5% (75/92) had a physical impairment, and 54.3% (50/92) had a nervous system disease; regarding the relatives, 81.5% (402/493) of the people with disabilities they were relatives of had a physical disability, 69.8% (344/493) had a mental disability, 66.3% (327/493) had a cognitive disability, and 36.7% (181/493) had a nervous system disease. Regarding the professionals, 30.8% (116/377) were rehabilitation professionals (not including physicians), 20.4% (77/377) were physicians, and 12.5% (47/377) were education professionals. A detailed description of the population had been previously published [18]. A total of 2018 open question answers about difficult life solutions for which a technical solution could be useful were analyzed automatically.

### Cluster Analysis

We conducted a clustering analysis of the answers about difficult life situations for which an innovative solution could be useful.

Table 1 presents the themes of the 14 clusters, their sizes, and internal consistencies. The cluster size varied from 9 to 681 answers. Five clusters (clusters 1 to 5 in Table 1), whose sizes were above a hundred answers, underwent a second step of clustering. Internal consistency varied from 42% to 100%. After reaching consensus, all κ values were above 0.85.

**Table 1.** Clustering analysis.

Main cluster and subcluster	Cluster theme	Internal consistency, % (n/N)
1		
1.1	To be able to^a^	91 (298/328)
1.2	Possibility through adapting^a^	58 (103/178)
1.3	Activities specific to children	48 (57/116)
1.4	Difficult situation in a given/specified location	59 (19/32)
1.5	Warmth and sociability^b^	54 (7/13)
1.6	Organization and scheduling	78 (7/9)
1.7	Behavioral disorders and mental disability	60 (3/5)
2		
2.1	To be able to do in daily life^a^	72 (114/158)
2.2	Difficulties with independence, need for support^a^	68 (50/73)
2.3	Friendship and social relations^b^	51 (19/37)
2.4	Children and youth relationships^b^	53 (18/34)
3		
3.1	Sports and activities	84 (109/130)
3.2	Impediments	83 (52/63)
4		
4.1	To be able to do (within a group or with help)^a^^,^^b^	80 (66/83)
4.2	Being able to do things “by myself”^a^	87 (55/63)
5		
5.1	Daily life, help and adaptation^a^	91 (68/75)
5.2	Travel issues, including technical aids and means	73 (51/70)
6	Action in the community^b^	66 (47/71)
7	Cluster of “doing,” everyday action	63 (36/57)
8	Unhappiness	54 (25/46)
9	Learning and autonomy^a^	42 (18/43)
10	Needed or proposed solution	70 (21/30)
11	Technological tools	58 (7/12)
12	Fine motor skills and communication	64 (7/11)
13	Marginal answers	100 (32/32)

a Clusters related to autonomy and desire for independence.

b Clusters referring to action within a group or community.

Cluster titles were defined by the 3 experts involved in the annotation process based on the dominant themes each identified as relevant, informed by their professional backgrounds and individual interpretations of the participants’ answers, and then an additional consensus decision was made, looking at the clinical, informational, and engineering understanding of each title that can allow sharing the results with decision-makers and solution developers.

Eight clusters highlighted difficulties related to autonomy and desire for independence*:* “To be able to,” “Possibility through adapting,” “To be able to do in daily life,” “Difficulties with independence, need for support,” “To be able to do (within a group or with a help),” “Being able to do things by myself,” “Daily life, help and adaptations,” and “Learning and autonomy.”

Four clusters highlighted difficulties related to actions within a group/community*:* “Warmth and sociability,” “Friendship and social relations,” “Children and youth relationships,” and “Actions in the community.”

Some clusters reinforced areas that emerged in the qualitative analysis, such as “Travel issues, including technical aids and means,” “Technological tools,” and “Sports and activities.”

### Frequency Analysis

**Figure 2.**
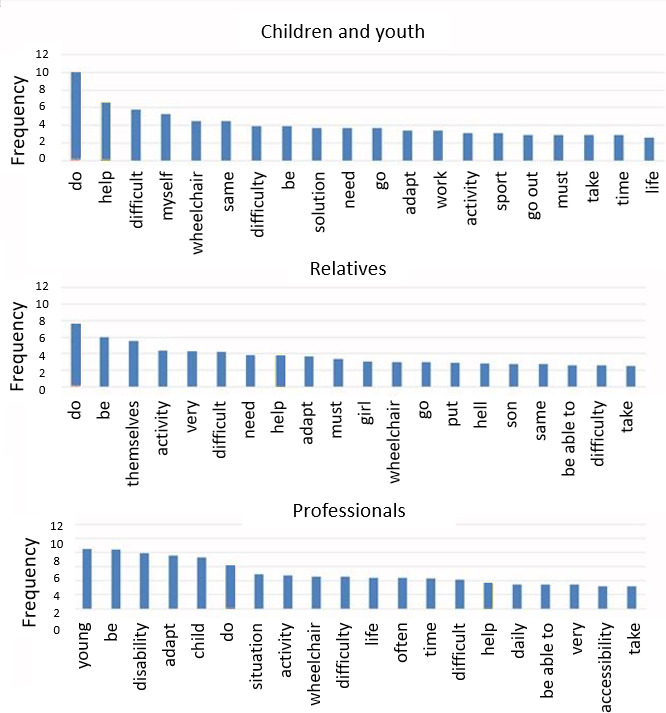
The first 20 most frequently quoted words by children and youth with disabilities, relatives, and professionals.

Figure 2 shows the frequency analysis of words for the 3 respondent groups. The most frequent occurrences differed across the groups. The term “do” was largely used by children and youth with disabilities and relatives (first position for both), closely followed by “myself/themselves” (respectively in fourth and third positions), underlying the need for technical solutions to facilitate autonomy. Other frequently used words were “help” (second position) and “difficult” (third position) for children and youth and “be” (second position) and “activity” for relatives. “Disability” was frequently used by professionals (third position) but did not figure among the 20 most frequently used terms by children and youth with disabilities and relatives. “Be” (second position) and “adapt” (fourth position) were also frequently used by professionals, whereas “do” appeared only in the sixth position.

The most frequent cooccurrence for the term “do” was “sport.” The most frequent cooccurrence for the term “help” was “human.”

It should be noted that high-frequency general terms such as “do,” “help,” and “be” are context dependent and can carry varied meanings across responses; for instance (examples from the responses): “doing sports,“doing pauses,” and “manage to do”; or “help my daughter to overcome her fear of water,” “human help,” and “adapted housing with human help”; “getting their school supplies out and doing homework without being helped by a parent,” “be frustrated,” and “be able to do.”

### Illustrative Quotes From Participant Responses

To illustrate how these terms were used in context, to clarify the conceptual distinctions between thematically similar clusters, and ground the analysis in the participants’ own words, response examples are presented in Table 2.

**Table 2.** Examples of participants’ answers to the survey according to the cluster they are related to.

Main cluster and subcluster	Theme	Examples^a^
1		
1.1	To be able to	To have an assistive robot for daily living that can anticipate and meet my needs as soon as I require help, so that I no longer have to rely entirely on my mother.^b^ Difficult travel/mobility has significant consequences on social ties, access to leisure, education, and employment.^c^ During a city center trip with four children in wheelchairs, we encountered bus transit issues; we were only allowed to board with two children. We had checked with the company beforehand, but they never mentioned a two-wheelchair maximum limit. While the company offers special assistance transport for disabilities, the application process is overly complicated, requiring an MDPH certificate proving a disability rating of over 80%.^d^
1.2	Possibility through adapting	Navigating outdoors or indoors (needing more effective navigation systems, particularly via GPS); computing/reading such as text-to-speech systems and Braille displays exist, but because current Braille displays only feature a single line, a multi-line display capable of rendering graphics (eg, on an A4-sized surface) would be incredibly useful.^b^
1.3	Activities specific to children	“Inappropriate games in the school playground: skipping ropes, elastic bands, and balls. Nothing but “physical” games.^b^" To design engaging games tailored for children with disabilities that able-bodied children would also want to play.^c^ Accessibility in schools and educational facilities is not always guaranteed, and access to adaptive sports still needs to be developed across many disciplines^d^
1.4	Difficult situation in a given/specified location	When visiting certain public spaces or shops downtown, there are very few access ramps, leaving parts of the town completely inaccessible to people with disabilities.^b^ Confined spaces, such as hotel rooms, where there is excessive noise (music, loud machinery, or maintenance tools).^c^ An indoor play center similar to Jim and Jump, but larger, more accessible, better suited, and catering to ages 0 to 20, as there is a severe lack of leisure facilities for people with disabilities.^c^
1.5	Warmth and sociability	To develop and foster social relationships for people with disabilities.^d^
1.6	Organization and scheduling	Creating a schedule using pictograms/establishing a routine for announcing daily schedules.^d^
1.7	Behavioral disorders and mental disability	Bringing in qualified external experts to explain behavioral disorders to care teams.^d^
2		
2.1	To be able to do in daily life	I have to exert deliberate effort for every single everyday task. For you, picking up a bottle of water is simple; for me, I have to focus on whether it’s empty or full and break the action down into smaller steps just to manage it, and this applies to everything. Eating, moving around, working... Because it’s a constant struggle, I get fatigued very easily.^b^ You constantly have to find someone to open doors to the buildings where tenants live; nothing is automated or accessible.^d^
2.2	Difficulties with independence, need for support	The hardest part for me is going shopping on my own, withdrawing cash... and dealing with accessibility issues around town.^b^ The terrain is too rugged to navigate unaided; operating the Joelette wheelchair requires at least two assistants.^d^
2.3	Friendship and social relations	His friends’ houses are rarely accessible.^c^ My son finds it hard to break out of his daily routine; the moment we do, it becomes incredibly difficult to bathe, feed, or put him to bed. Consequently, it’s highly challenging (if not impossible) for us to go away for the weekend or spend an evening at a friend’s house.^c^
2.4	Children and youth relationships	Due to her hemiparesis, she is not yet able to get dressed on her own. Although she is eager to join a club despite her young age, she feels different from her peers and has chosen to step back.^c^ He asks to invite friends over, but when they arrive, he seems happy yet unable to actually play with them; he just shares his snack, and we end up having to supervise both children.^c^
3		
3.1	Sports and activities	Adapting the PS4 controller. Not being able to use it means I can’t play video games with my friends at the youth center.^b^ As an adaptive physical activity teacher, I reach out to as many organizations as possible to ensure sports are integrated into school programs. The challenges we still face involve facility accessibility and a lack of understanding among support staff. We constantly have to justify why regular physical activity is beneficial for these children; it is an uphill battle. Furthermore, financial support for adaptive sports equipment should be automatic, as the support network in this area is currently underdeveloped.^d^ Designing a finger prosthesis with a specific orientation that reduces instrument weight (for agenesis); creating arm protection sleeves and jacket fastenings to keep the upper limb from swinging unprompted (for fencing with brachial plexus palsy).^d^
3.2	Impediments and barriers	It is difficult to find accessible holiday destinations.^b^ Difficulty navigating the city and ongoing accessibility issues.^d^
4		
4.1	To be able to do (within a group or with help)	Typing a text on a smartphone with one hand. Cutting a piece of meat. Playing team sports...^b^ I have previously organized a play session in a park for a 4-year-old alongside other children.^d^ Ultimately, it all comes down to how well parents can tolerate the judgment and stares of others! Going for a walk or doing activities with a child who is different can provoke uncomfortable reactions or outright judgment.^d^
4.2	Being able to do things “by myself”	Having to wait a long time to use the restroom until a professional is available to help.^b^ He is completely dependent on others. He loves going out into nature, meeting people, and listening to music, but he cannot do any of it on his own.^c^ She cannot climb play structures with ropes and ladders by herself.^c^
5		
5.1	Daily life, help, and adaptation	For every single aspect of daily life, you have to constantly encourage, guide, and supervise.^c^ There are no suitable adult changing facilities in public spaces.^c^
5.2	Travel issues, including technical aids and means	Navigating uneven terrain in a standard wheelchair, or flying when a person cannot sit upright (a major obstacle for families with relatives abroad, especially nowadays when air travel is so common). We need an airline-approved, highly portable system (like a specialized harness or a mechanism to secure the seat shell of an adaptive stroller).^d^
6	Action in the community	Creating/adapting board games.^c^ Yes, for dancing, she absolutely loves it. We engineered a custom harness that allows her to dance standing up rather than always being confined to her seat.^c^
7	Cluster of “doing,” everyday action	Styling my hair into a ponytail; washing myself; raising my arm.^b^ Tying shoelaces; holding a knife and fork.^c^ Going grocery shopping.^c^
8	Unhappiness	Severe frustration surrounding sexuality and intimacy^b^ Sleep disrupted by chronic back pain.^b^
9	Learning and autonomy	Training programs for local stakeholders and community members.^d^ Educational and instructional support provided by trained staff.^d^
10	Needed or proposed solutions	I haven’t found any viable, long-term solutions. Just a few basic tools, like visual aids.^b^ Correct equipment installation and assistive switches/contactors.^d^
11	Technological tools	Robotic tools and artificial intelligence.^d^ Human dependence on IT tools and robotics.^d^
12	Fine motor skills and communication	Implementing the Makaton vocabulary system.^c^ Hand-writing text.^b^

a Translations from French into English.

b A response from a child or youth with disabilities.

c A response from a relative.

d A response from a professional.

While several clusters share an autonomy theme, they differ in their perspectives. Cluster 2.1 includes broad, general daily life activities, whereas cluster 4.2 encompasses expressions of an explicit, first-person desire for independence, and self-empowerment. Cluster 7 comprises concrete and highly localized motor and routine acts of execution. Similarly, community clusters (1.5, 2.3, 2.4, and 6) vary in relational scope, ranging from specific peer friendships to broader social integration.

## Discussion

### Principal Findings

Text mining has indicated the significant role of autonomy and the theme of “doing it by myself.” This theme appears to be enriched through computational analysis compared to prior human-led qualitative analysis. This point suggests that both approaches can and are complementing one another. Thus, our study suggests that there is a need for innovative solutions that can facilitate autonomy and the ability to do things independently and facilitate integration into groups and community life.

The findings underscore the importance and the need of developing solutions addressing mobility, travel, and sports needs [18]. Additionally, the analysis also revealed differences in language across the 3 respondent groups. “Do” and “by myself” ranked among the 5 most frequent words for children and youth with disabilities and relatives. “Disability” was among the most frequent words used by professionals but was not among the 20 most frequent words used by the other respondent categories (eg, children and youth with disabilities and relatives).

### Actionable Knowledge Uncovered by Text Mining Analysis

The need for solutions promoting autonomy and self-empowerment was highlighted by both the frequency and clustering analyses, echoing previous work in the literature on the role of autonomy in parental well-being [51] and the importance of evaluating the impact of assistive devices on autonomy [52]. Additionally, frequency analysis revealed that autonomy and self-empowerment were frequently mentioned by children and youth with disabilities and relatives but less frequently by professionals. In contrast, professionals more frequently mentioned the concept of adaptation (“adapt”) than the concept of action (“do”). First, these text mining results highlighted a desire for autonomy, a theme less prominently amplified in the human-led qualitative analysis, which the text mining approach detected through a computational and less expectation-biased analytical lens [18]. Interestingly, a comparison of human-led and large language model (LLM)–assisted approaches to analyzing the narratives of people with cancer also found that text mining analysis highlighted the active role of patients, with ChatGPT-4o emphasizing patients’ proactive use of digital platforms to gather medical information and manage appointments [29]. Human coders—who were here researchers and physicians—may have their own biases. Humans are inevitably influenced by personal perspectives and theoretical frameworks [29]. Therefore, our results reinforced the complementarity between human-led qualitative analysis and text mining to uncover the desire for autonomy and the ability to “do it by myself.” Second, these results demonstrated the importance of taking into account the points of view of various stakeholders to build as complete a picture of needs as possible, as previously reported [16,17,52]. This type of approach may also help professionals adjust their proposals to children and youth with disabilities’ and relatives’ concerns [36].

Regarding patients’ preferences for the way physicians communicate with them, it has been shown that they prefer their physicians to avoid medical jargon [53]. Interestingly, it appears that even a common language term such as “disability” was frequently used by the professionals but not by the children and youth with disabilities or the relatives. On the contrary, children and youth with disabilities and relatives emphasize action-oriented language such as “do.” These results suggest that children and youth with disabilities and their relatives prioritize action. This should be taken into account by health professionals in the process of shared decision-making with their patients. Furthermore, based on these results, further studies could evaluate the preferences of children and youth with disabilities and relatives about words they want to be chosen or avoided by their professionals when evaluating a life situation, similar to a study that specifically evaluated person-first and identity-first language preferences for various chronic conditions [54].

The emergence of multiple clusters that share an action-oriented or autonomy-related theme allowed to approach the subject from different perspectives and therefore to deepen overall understanding of these topics. While cluster 2.1 (“To be able to do in daily life”), cluster 4.2 (“Being able to do things by myself”), and cluster 7 (“doing, everyday action”) all center on autonomy, they shed light on the subject from a different angle. Cluster 2.1 included broad, general daily life tasks and situational activities across a wide spectrum of daily functions. Cluster 4.2 encompassed expressions of an explicit, first-person desire for independence and self-empowerment. Cluster 7 comprised concrete motor and routine acts of execution characterized by concrete everyday verbs and actions such as “to wash” or “shoelace.” Recognizing these distinctions prevents the oversimplification of “autonomy” into a single homogeneous concept, highlighting instead that user needs span from physical task execution to overarching psychological independence. However, this did not rule out some overlap between clusters; some responses classified under a given theme might also have corresponded to another theme within the same group (autonomy related or action related).

The need for solutions to facilitate integration into community life was also revealed by the clustering analysis. Several clusters reinforced this point, offering insights that deepened understanding of this need. The parents of children with a disability often face difficulties in their social interactions [55]. Our results suggested technical solutions could alleviate some of those difficulties and facilitate broader integration into community life. This aligns with the findings of a scoping review showing that technical issues for robots that constitute promising opportunities for inclusive classrooms should be overcome for their effective use [7]. Our results suggest that similar needs and opportunities to those raised in the school context are relevant across a wider range of social contexts.

### Reinforcement of Knowledge From Qualitative Analysis

Comparison of the results of the text mining analysis with those of the human-led qualitative analysis of answers to the open questions [18] confirmed and reinforced the need for innovative solutions to overcome difficulties in the following domains: mobility and accessibility (reinforced by the clustering analysis), sports (reinforced by the clustering analysis, frequency analysis, and analysis of cooccurrence), and high-technology devices (reinforced by the clustering analysis). The frequent association of the terms “help” and “human” echoed the previously highlighted need for solutions to support relatives. This cooccurrence reinforces an important conceptual point: assistive technologies are not intended to replace human assistance but to complement and facilitate it. The aspiration for autonomy expressed by participants coexists with an acknowledgment of the continuing value of human support, as a balance between “high-tech” and “high-touch” care that should guide both the design of assistive technologies and their integration into the daily lives of individuals with disabilities and their families.

### Limitations

This study has several limitations. First, it was based on an online survey; therefore, internet access was required to respond. This may have induced a selection bias. However, an online survey allows a large range of people to respond. More than 1000 answers were obtained; however, some respondents did not complete the optional open questions. Therefore, analysis by respondents’ categories was possible for the frequency analysis but not the clustering.

Internal consistency was insufficient for exhaustive classification of each answer, but enabled 40% to 100% of consistent answers to be grouped, allowing meaningful knowledge to emerge. Therefore, the clustering analysis effectively reinforced some findings highlighted by the human-led qualitative analysis and uncovered new findings, rather than exhaustively classifying answers. The difficulty of classifying items into strict emerging themes has recently been highlighted by Prescott et al [28] in a study comparing human-led and LLM-based analyses of English text messages. They evaluated theme overlap between both methods at approximately 70%. However, intercoder reliability between LLM and humans, operationalized as the number of agreements in coding divided by the sum of agreements and disagreements in coding, varied between ~40% to 50% and thus showed results comparable to ours.

Some specific issues may also make it difficult to achieve higher internal consistency. This may be first linked to the few existing developments in clustering the French language. Furthermore, some answers included explanations of multiple topics within a single response, resulting in overlap. This could be resolved by manually splitting answers that cover different topics before the analysis; however, that is a time-consuming process. Additionally, because TF-IDF operates at the individual word level (ie, unigrams), multiword expressions and frequent collocations (eg, “do sports,” “go outside”) are not captured as meaningful units; this may limit the model’s ability to reflect the full semantic content of participants’ responses. Incorporating bigram or trigram features in future analyses or using algorithms such as Word2Vec [56] or topic modeling [38] could address this limitation and may also improve the internal consistency of clusters with higher thematic overlap, such as cluster 9 “learning and autonomy.” To improve data gathering in the future, the online survey materials could be adapted to include the option of describing issues across different fields; this was not available with the free tool we used. Furthermore, differences in internal consistency across researchers may be partly explained by subjectivity induced by background differences. However, the different backgrounds may help to reach a consensus and avoid bias.

### Future Work

Clustering may be facilitated by automated indexing using existing ontologies [57-59]. The International Classification of Functioning, Disability, and Health (ICF) framework [5] could be considered for this purpose; however, the ICF is a conceptual and technical description of daily life and participation. As such, the use of ICF terminology may not correctly label the everyday language of nonprofessional respondents, whose opinions are crucial in this context. This issue is recurrent in health care data, which are often heterogeneous. Integrating various ontologies into models to combine data across different technical contexts, levels of language, and levels of data structuration [60] remains a challenge, even for English-language data. One group [60] annotated a corpus and created dictionaries to analyze Chinese posts on a questions and answers forum about rehabilitation issues using BERT-BiGRU-attention models. The posts included questions from patients and answers from physicians, and therefore, data with different levels of language and technicity, although the questions and answers were strongly contextually linked [60]. The analysis led to the creation of a relationship dictionary of diseases and their corresponding symptoms. Our results could also be used to enrich existing ontologies [61] to facilitate the integration of heterogeneous data on daily life and participation. This enrichment would support text data analysis across different levels of technicity and incorporate the perspectives of diverse stakeholders, such as health care professionals, education professionals, relatives, and children and youth with disabilities.

The next step to enhance clustering performance could involve integrating LLMs into the process [62,63]; however, these models may reproduce bias from their training data [30]. Therefore, care must be taken when applying LLMs to heterogeneous datasets—including answers from individuals with disabilities, relatives, and professionals—to ensure that the model is not biased toward a particular linguistic or technical level. More broadly, the use of LLMs in qualitative health research raises ethical questions regarding algorithmic bias and representational fairness. LLMs trained predominantly on majority-language, able-bodied corpora risk underrepresenting the voices of individuals with disabilities and may reproduce institutional framings of disability that diverge from the action-oriented language used by children and youth with disabilities and relatives in this study. Transparency in model selection, systematic evaluation of outputs for differential performance across participant groups, and human oversight at the interpretation stage are therefore essential safeguards for any future LLM-assisted analysis of this type of dataset.

From an application perspective, such an approach may help prioritize the development of assistive technologies for people with disabilities. However, the perceived limited market for assistive products and technologies results in high selling prices due to development costs. Development needs should be organized by user profile rather than by the same expressed need. Clustering development needs could support the identification of opportunities to extend an existing solution to a related need, regardless of its original domain, or to highlight needs that represent a sufficiently large market for new solution development. For this aim, such an analysis may be repeated at regular intervals, in France, as well as in other countries with different public health policies.

### Conclusions

Analysis of 2018 open-ended answers using a text mining approach designed for French data on needs in terms of activity limitations and participation restrictions for which assistive products and technology may be useful for children and youth with disabilities revealed that solutions are required to facilitate autonomy and the ability to “do it myself (or themselves)” as well as integration into group and community life. The results reinforced the need for solutions to facilitate mobility, travel, and sports, as highlighted by the human-led qualitative analysis. Combining text mining analysis with human-led qualitative analysis may help prioritize solution development by identifying recurrent needs. The text mining analysis also highlighted differences in language use across the 3 respondent groups, demonstrating the importance of questioning all stakeholders to build a comprehensive picture, and suggesting that professionals should consider the terms they use with children and youth with disabilities and relatives.

## Abbreviations

EACD: European Academy of Childhood Disability
ICF: International Classification of Functioning, Disability, and Health
LLM: large language model
SFERHE: Société Francophone d'Etudes et de Recherche sur les Handicaps
TF-IDF: Term Frequency–Inverse Document Frequency
